# Calf Thymus Polypeptide Restrains the Growth of Colorectal Tumor *via* Regulating the Intestinal Microbiota-Mediated Immune Function

**DOI:** 10.3389/fphar.2022.898906

**Published:** 2022-05-19

**Authors:** Lanzhou Li, Chenfei Zhao, Fange Kong, Yi-Cong Li, Chunxia Wang, Shanshan Chen, Hor-Yue Tan, Yang Liu, Di Wang

**Affiliations:** ^1^ Engineering Research Center of Chinese Ministry of Education for Edible and Medicinal Fungi, Jilin Agricultural University, Changchun, China; ^2^ School of Life Sciences, Jilin University, Changchun, China; ^3^ Centre for Chinese Herbal Medicine Drug Development, School of Chinese Medicine, Hong Kong Baptist University, Hong Kong, China

**Keywords:** calf thymus polypeptide, colorectal cancer, intestinal microbiota, T cells, NK cells, interleukin-2

## Abstract

Calf thymus polypeptide (CTP), with a molecular mass of <10 kDa, is prepared from the thymus of less than 30-day-old newborn cattle. In the present study, the inhibitory function of CTP in colorectal cancer (CRC) was investigated in B6/JGpt-*Apc* ^*em1Cin(MinC)*^/Gpt (*Apc* ^Min/+^) mice. CTP hampered tumor development and enhanced the ratio of CD3e^−^NK1.1^+^ cells by 113.0% and CD3e^+^CD28^+^ cells by 84.7% in the peripheral blood of *Apc* ^Min/+^ mice. CTP improved the richness, diversity, and evenness of the intestinal microbiota of *Apc* ^Min/+^ mice, particularly by regulating the abundance of immune-related microorganisms. CTP effectively regulated the expression of immune-related cytokines, such as interleukin (IL)-2 (15.19% increment), IL-12 (17.47% increment), and transforming growth factor (TGF)-β (11.19% reduction). Additionally, it enhanced the levels of CD4 and CD8, as well as the ratio of helper T lymphocytes (Th)1/Th2 in the spleen and tumors of *Apc* ^Min/+^ mice. In CTP-treated mice, reduced levels of programmed death-1 (PD-1), programmed cell death-ligand 1 (PD-L1), cytotoxic T lymphocyte-associated antigen 4 (CTLA4), activated nuclear factor of activated T cells 1 (NFAT1), and nuclear factor κB (NF-κB) p65 signaling were noted. Collectively, the anti-CRC effect of CTP is related to the modulation of intestinal microbiota-mediated immune function, which provides a reference for CTP as a therapeutic drug or a combination drug used in CRC treatment in a clinical setting.

## 1 Introduction

Colorectal cancer (CRC) is a disease prevalent globally, resulting in a serious health burden, which is the fourth most common cancer (age-standardized prevalence: 19.7/100,000) and the third cause of cancer deaths (age-standardized mortality: 9/100,000) worldwide (Schliemann et al., 2021). Most CRC patients have no significant genetic correlation, and diet, age, and living habits may be key driving factors for the occurrence and development of CRC (Wang et al., 2018; Schulpen and van den Brandt, 2020). According to the location and progression of the tumor, standard treatments for CRC include surgery, chemotherapy, radiotherapy, and combined treatment. It is almost impossible to completely eliminate cancer cells using surgery alone (Miller et al., 2019). The 5-year survival rates following surgical removal of CRC tumors are 91.1% (localized), 71.7% (regional), and 13.3% (distant) in the United States, respectively (Siegel et al., 2017). More than 60% of CRC regional patients received further treatment with adjuvant chemotherapy and/or radiotherapy (Miller et al., 2019). Even with neoadjuvant (Chemo) chemoradiotherapy, local tumor recurrence occurs in 10% of patients with CRC (Ogura et al., 2019). Therefore, it is crucial to develop alternative, safe, and efficacious treatment strategies for patients with CRC.

Immune responses recognize cancer cells and potentially eradicate tumors (Gajewski et al., 2013). Activated T cells promote immune response by secreting interferon (IFN)-γ (Mandai et al., 2016), or directly killing tumor cells by cytotoxicity (Su et al., 2018). Tregs inhibit the immune effect of T cells through the programmed death-1 (PD-1)/programmed cell death-ligand 1 (PD-L1) combination (Khaja et al., 2017). PD-1, expressed on the surface of T cells, binds to Tregs or tumor cells through its ligand PD-L1, resulting in a weakened immune response (Kollmann et al., 2017; Langhans et al., 2019). Accordingly, PD-1 and PD-L1 antibodies show dynamic and lasting tumor regression effects in a clinical setting (Sunshine and Taube, 2015). Direct administration of interleukin (IL)-2 or adoptive treatment of T cells cultured in the presence of IL-2 is one of the earliest curable systemic therapies for all solid tumors (Rosenberg, 2014).

Conversely, the intestinal microbiota and their products remarkably affect immune function (Zhou et al., 2020). The occurrence and development of CRC are related to a decrease in beneficial bacteria, such as *Lactobacillus*, and an increase in pathogenic bacteria, such as *Fusobacterium* (Lenoir et al., 2016; Yamaoka et al., 2018). *Desulfovibrio* species are linked to the incidence of ulcerative colitis (Rowan et al., 2010). Short-chain fatty acids in the secondary metabolites of the intestinal microbiota help regulate the activation of innate immune cells and inhibit the pathological progression of CRC (Ma et al., 2019; Tarashi et al., 2019).

In 1965, thymosins were firstly extracted from the bovine thymus (Hannappel, 2003). Thymosins have been reported to show effects such as increasing tissue repair and regeneration and regulating immune cell function and development (Garaci et al., 2015; Goldstein and Kleinman, 2015). In China, calf thymus polypeptide (CTP) has been approved for clinical application (Chinese national medicine permission number: H22025973 for injection and H20065576 for tablets) and augments the quality of life and survival rate of cancer patients when administered in combination with chemotherapy (Garaci et al., 2015; Zeng et al., 2019). In our previous research, it has been confirmed that CTP effectively improves natural killer (NK) cells’ killing activity and lymphocyte transformation activity in immunosuppressive mice, and significantly improves the hematopoietic function via modulation of colony-stimulating factors in mice with hematopoietic dysfunction; CTP can also enhance the levels of IL-2 in the serum and spleen (Li et al., 2020). However, the direct anti-tumor effect of CTP has not yet been reported.

Adenomatous polyposis coli (APC) gene mutation is the starting point of 80% of sporadic CRC (Zeineldin and Neufeld, 2013), and most sporadic CRC patients lose two APC alleles (Kinzler and Vogelstein, 1996). *Apc* ^Min/+^ transgenic mice are considered a classic model of CRC (Zeineldin and Neufeld, 2013). In the present study, we determined the inhibitory effect of CTP on CRC in B6/JGpt-*Apc* ^em1Cin(MinC)^/Gpt mice by regulating intestinal microbiota and their immune response.

## 2 Materials and Methods

### 2.1 CTP Preparation

CTP, a multi-component peptide, was prepared by hydrolyzing and ultrafiltration from less than 30-day-old newborn cattle thymus (provided by Jilin Connell Pharmaceutical Co., Ltd., Jilin, China) similar to our previous study (Li et al., 2020);17.73% of CTP has a molecular mass of 1,000–10,000 dalton (D) and 82.27% has a molecular mass of <1,000 D. CTP contains 17 types of amino acids, and the detailed information can be found in our previous study (Li et al., 2020).

### 2.2 Protocol for Animal Experimentation and Drug Administration

Eight-week-old male B6/JGpt-*Apc* ^*em1Cin(MinC)*^/Gpt (*Apc* ^Min/+^) mice (genotype: APC; genetic background: C57BL/6JGpt; heterozygous) (25 ± 1 g, specific pathogen-free (SPF) grade, SCXK (SU) 2018–0009), purchased from GemPharmatech Co., Ltd. (Jiangsu, China), were housed in a controlled environment at 23 ± 1°C and humidity 55 ± 5% under a 12/12-h light/dark cycle (lights on from 7:00 a.m. to 7:00 p.m.). Sterilized high-sugar and high-fat diets (D12492; 60% kcal fat, 20% kcal protein, and 20% kcal carbohydrate; Xiao Shu You Tai Biotechnology Co., Ltd., Beijing, China) and water were provided *ad libitum*. The experimental protocol complied with ARRIVE guidelines and was approved by the Institution Animal Ethics Committee of Jilin University (No. SY201910003).

After 1 week of adaptive feeding, 18 male mice were randomly divided into two groups and intraperitoneally (i.p.) received 0 mg/kg (*n* = 9) (vehicle-treated mice) and 6.75 mg/kg of CTP (*n* = 9) (CTP-treated mice) once per day for 56 days; body weights were recorded on the days 1, 14, 28, 42, and 56. Blood samples were collected from the caudal veins of the mice 1 h after the last administration. The mice were then euthanized by CO_2_ inhalation, and their colorectum with cecum and anus was carefully dissected; cecum contents were collected under aseptic conditions and stored at −80°C for intestinal microbiota analysis. After cleaning the colorectum with normal saline, its weight and length were analyzed; the colorectal index was calculated as follows:

Colorectal  index(g/m)=colorectal weight(g)/colorectal  length(m).

The heart, liver, spleen, lung, thymus, and kidney specimens of each mouse were collected and weighed, and organ coefficients were calculated as follows:

Organ index (mg/g)=organ weight (mg)/body weight (g).

### 2.3 Peripheral Blood Component Assay

Three peripheral blood samples from each group were randomly selected, and white blood cells (WBC), lymphocytes (LYM), monocytes (MON), granulocytes (GRA), red blood cells (RBC), mean corpuscular volume (MCV), platelets (PLT), and mean platelet volume (MPV) were analyzed using a fully automated blood analyzer (Drew Scientific Inc., Oxford, CT, United States).

### 2.4 Measurement of NK and T Cells in the Peripheral Blood Using Flow Cytometry

The non-nucleated cells of six peripheral blood samples (randomly selected) from each group were removed using a red blood cell lysis buffer (00-4333-57), and residual cells were re-suspended in cell staining buffer (00-4222-26) to adjust the cell concentration to 1 × 10^7^/ml. A 100 μl cell suspension from each sample was coated with NK cells (CD3e^−^NK1.1^+^) with fluorescein isothiocyanate (FITC)-conjugated anti-CD3e monoclonal antibody (11-0031-82) and allophycocyanin-conjugated anti-NK1.1 antibody (17-5941-82) or coated for activated T cells (CD3e^+^CD28^+^) with FITC-conjugated anti-CD3e monoclonal antibody (11-0031-82) and allophycocyanin-conjugated anti-CD28 antibody (17-0281-82) for 15 min at 25 ± 1°C. FITC-conjugated anti-Armenian hamster IgG (11-4888-81), allophycocyanin-conjugated anti-mouse IgG2a (17-4724-81), and allophycocyanin-conjugated anti-Syrian hamster IgG (17-4914-81) were used as the isotype controls. The cells were then analyzed using a CytoFLEX flow cytometer according to the manufacturer’s instructions. All antibodies and buffers were obtained from eBioscience (San Diego, CA, United States).

### 2.5 Histopathological Analysis and Immunohistochemical Examination

The colorectum, heart, liver, spleen, kidney, and lung specimens collected from *Apc* ^Min/+^ mice were fixed in 4% paraformaldehyde for 48 h. Samples were embedded in paraffin, sliced into 5 μm sections, and stained with hematoxylin and eosin (H&E), as described in a previous study (Elsherbiny et al., 2017).

Other sections of the colorectum (especially the colorectal tumor portion) and spleen were blocked with 5% bovine serum albumin (Gen-view Scientific, Galveston, TX, United States ) for 4 h and incubated with primary antibodies including CD4 (25229S, 1:200 dilution) and CD8 (98941S, 1:800 dilution) (Cell Signaling Technology, Danvers, MA, United States ), IFN-γ (PA5-95560, 1:1000 dilution) and IL-4 (PA5-25165, 1:50 dilution) (Invitrogen, Carlsbad, CA, United States ) at 4°C overnight, followed by incubation with horseradish peroxidase (HRP)-labeled secondary antibodies (E-AB-1003 or E-AB-1001) (Elabscience, Wuhan, China) at 4°C for 2 h. Color development was performed using the Metal Enhan DAB Substrate Kit (34,065, Thermofisher, Carlsbad, CA, United States ), and hematoxylin was used for counterstaining. All the slides were observed under a light microscope (Olympus Corporation, Tokyo, Japan). ImageJ software (National Institutes of Health, Bethesda, MD) was used to quantify the pixel density for the semi-quantitative densitometric analysis of protein expressions.

### 2.6 Intestinal Microbiota Analysis

The contents from five cecum samples collected from vehicle-treated mice and four cecum samples collected from CTP-treated mice were selected randomly for 16S rRNA gene analysis of the intestinal microbiota. The total mass of microbial DNA ranging from 1.2 to 20.0 ng isolated from each cecum content using the OMEGA Soil DNA Kit (M5635-02) (Omega Bio-Tek, Norcross, GA, United States ) was stored at −20°C. The forward primer 338F (5′-ACT​CCT​ACG​GGA​GGC​AGC​A-3′) and reverse primer 806R (5′-GGACTACHVGGGTWTCTAAT-3′) were used to amplify the V3–V4 regions of the bacterial 16S rRNA gene by PCR amplification. The amplicons were pooled in equal amounts, and pair-end 2 × 250 bp sequencing was performed using the Illumina MiSeq platform with a MiSeq Reagent Kit v3 at Shanghai Personal Biotechnology Co., Ltd. (Shanghai, China). The bacteria sequences were uploaded to the NCBI Sequence Read Archive with the accession number PRJNA796779 (https://www.ncbi.nlm.nih.gov/sra/PRJNA796779). This analysis was performed as described in our previous study (Jiang et al., 2021).

### 2.7 Assay of Cytokines in the Serum and Colon of *Apc* ^Min/+^ Mice

The levels of IL-1β (FY2040-A), IL-2 (FY2698-A), IL-6 (FY2163-A), IL-8 (FY2123-A), IL-10 (FY2176-A), IL-12 (FY2105-A), immunoglobulin (Ig) A (FY2055-A), IgG (FY2057-A), toll-like receptor (TLR) 4 (FY2816-A), TLR5 (FY30131-A), and transforming growth factor (TGF)-β (FY2686-A) (Jiangsu Feiya Biological Technology Co., Ltd., Jiangsu, China), and IL-4 (EK0405) (Wuhan Boster Biological Engineering Co., Ltd., China, Wuhan, China) in the serum and colon were determined using enzyme-linked immunosorbent assay (ELISA) kits in accordance with the manufacturer’s instructions in *Apc* ^Min/+^ mice.

### 2.8 Western Blot

The spleen and colorectal tumor tissues collected from *Apc* ^Min/+^ mice were lysed with radioimmunoprecipitation assay lysis buffer containing a 1% protease inhibitor cocktail (Sigma-Aldrich, St. Louis, MO, United States) and 2% phenylmethanesulfonyl fluoride (Sigma-Aldrich, St. Louis, MO, United States). The protein content was analyzed using a bicinchoninic acid protein assay kit (Merck, Darmstadt, Germany). The obtained proteins (40 µg) were separated by 10%–12% sodium dodecyl sulfate polyacrylamide gel electrophoresis and transferred onto 0.45-μm polyvinylidene difluoride membranes (Merck, Darmstadt, Germany). The membranes were blocked with 5% bovine serum albumin (Gen-view Scientific, Galveston, TX, United States ) at 4°C for 4 h and then incubated with primary antibodies including CD4 (bs-0647R, 1:1000 dilution), CD8 (bs-0648R, 1:1000 dilution), PD-1 (bs-1867R, 1:1000 dilution), PD-L1 (bs-4941R, 1:1000 dilution), cytotoxic T lymphocyte associated antigen 4 (CTLA4) (bs-10006R, 1:1000 dilution) (Bioss, Beijing, China), nuclear factor of activated T cells 1 (NFAT1) (ab49161, 1:800 dilution), calcineurin A (ab109412, 1:10000 dilution), protein kinase B (AKT) (ab200195, 1:2000 dilution), phosphor (P)-AKT (ab108266, 1:2000 dilution) (Abcam, Cambridge, MA, United States ), inhibitor of nuclear factor kappa-B kinase α/β (IKKα/β) (A2062, 1:1000 dilution), P-IKKα/β (AP0891, 1:1000 dilution), nuclear Factor κB (NF-κB) p65 (A19653, 1:1000 dilution), P-NF-κB p65 (AP0475, 1:500 dilution), IL-2 (A16317, 1:1000 dilution) (Abclonal, Wuhan, China), phosphatidylinositol 3-kinase (PI3K) (4263, 1:1000 dilution), P-PI3K (4228, 1:1000 dilution) (Cell Signaling Technology, Danvers, MA, United States ), and glyceraldehyde-3-phosphate dehydrogenase (GAPDH) (ABS16, 1:1000 dilution) (Millipore, Merck, Darmstadt, Germany) overnight at 4°C. After washing with 0.1% Tween-20 Tris-buffer, the membranes were exposed to horseradish peroxidase (HRP)-conjugated secondary antibodies (E-AB-1001 or E-AB-1003) (Elabscience, Wuhan, China) at 4°C for 4 h. Protein bands were developed using an enhanced chemiluminescence detection kit (Merck, Darmstadt, Germany) and visualized using the BioSpectrum 600 imaging system. The pixel density was measured using ImageJ software (National Institutes of Health, Bethesda, MD, United States).

### 2.9 Statistical Analysis

All values are expressed as mean ± standard deviation (S.D.). A one-way analysis of variance (ANOVA) was performed to detect the statistical significance using SPSS 19.0.0 software (IBM Corporation, Armonk, NY, United States). A *p*-value < 0.05 was considered statistically significant.

## 3 Results

### 3.1 CTP Suppressed CRC Growth in *Apc* ^Min/+^ Mice

**FIGURE 1:**
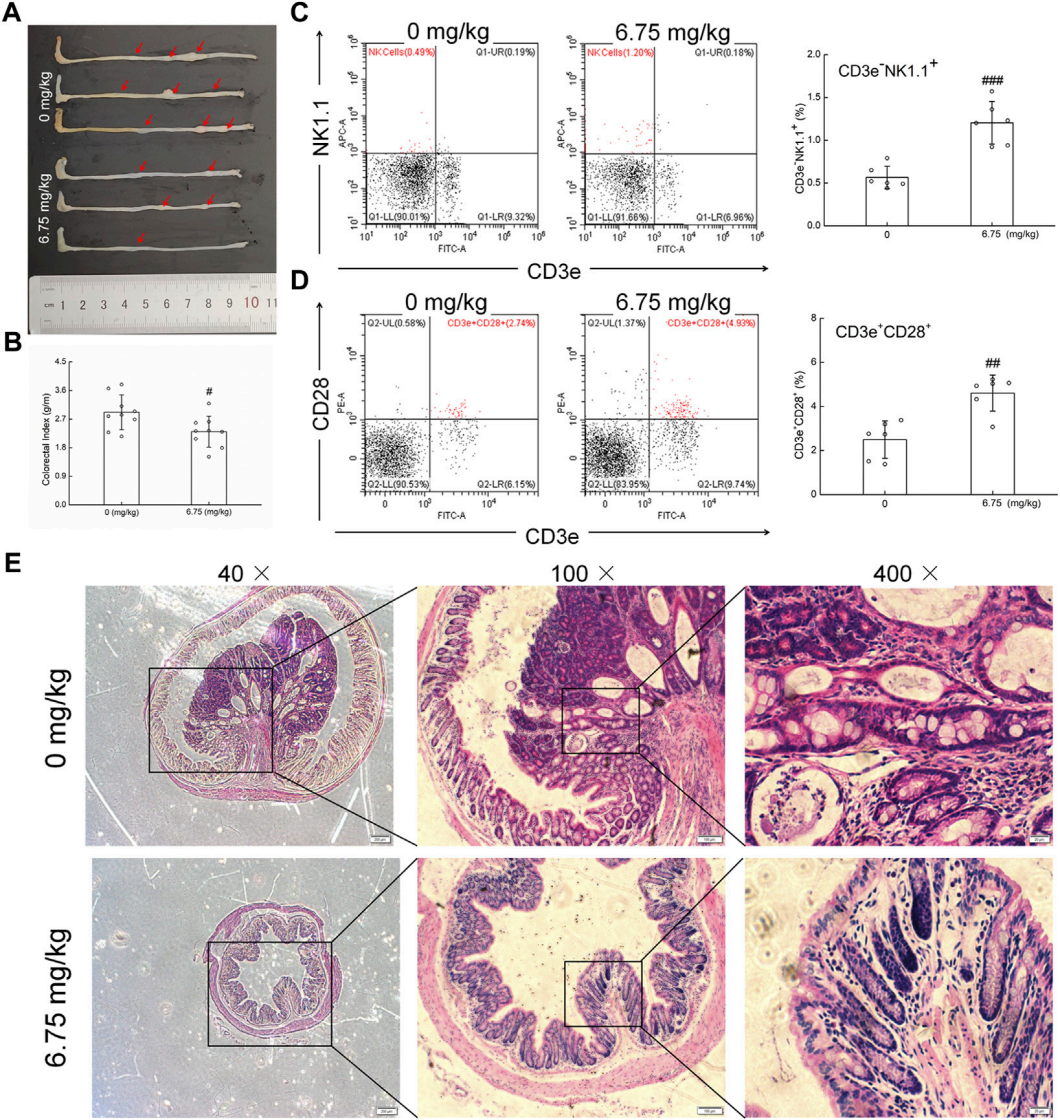
Anti-CRC effect of CTP on *Apc* ^Min/+^ mice. **(A)** CTP inhibited the development of CRC in *Apc* ^Min/+^ mice. **(B)** CTP reduced the colorectal index in *Apc* ^Min/+^ mice (*n* = 9). **(C)** CTP enhanced the levels of NK cells (CD3e^−^NK1.1^+^) in the peripheral blood of *Apc* ^Min/+^ mice (*n* = 6). **(D)** CTP enhanced the levels of T cells (CD3e^+^CD28^+^) in the peripheral blood of *Apc* ^Min/+^ mice (*n* = 6). **(E)** Histopathological observation of colorectal tumor in *Apc* ^Min/+^ mice (40 ×, scale bar: 200** **μm; 100 ×, scale bar: 100 μm; and 400 ×, scale bar: 20 μm) (*n* = 3). Data are presented as mean ± S.D. and analyzed via a one-way ANOVA test. ^#^ *p* < 0.05, ^##^ *p* < 0.01, and ^###^ *p* < 0.001 vs. vehicle-treated mice.

In the colorectum of *Apc* ^Min/+^ mice, an obvious colorectal tumor formation was noted. In contrast, CTP strongly suppressed the number and size of colorectal tumors (Figure 1A) and decreased the colorectal index by 20.98% (*p* < 0.05) (Figure 1B) without affecting the body weight and organ indices, except for the spleen (42.49% increment vs. vehicle-treated mice) (Table 1). The development of colorectal proliferative tissue in *Apc* ^Min/+^ mice was significantly inhibited by CTP, indicating a more normal morphology of intestinal epithelial cells and a more obvious infiltration of immune cells in CTP-treated mice than in vehicle-treated mice (Figure 1E). No significant changes in the pathological sections of the heart, liver, spleen, and lungs were noted in either vehicle- or CTP-treated *Apc* ^Min/+^ mice (Supplementary Figure S1).

**TABLE 1:** Effect of CTP on the body weight and organ index of *Apc* ^Min/+^ mice.

		0 mg/kg	6.75 mg/kg
Body weight (g)	1^st^ day	26.9±1.6	26.2±1.2
	14^th^ day	28.1±1.9	28.3±1.9
	28^th^ day	28±3.3	27.8±2.7
	42^nd^ day	25.6±2.5	26.8±3.3
	56^th^ day	23.2±3.2	22.0±2.4
Organ index (mg/g)	Heart	9.4±1.6	10.1±2.4
	Liver	50.8±6.4	53.9±5.8
	Spleen	11.4±4.1	16.2±3.4^#^
	Lung	8.1±2.2	7.6±0.7
	Kidney	12.7±0.5	13±0.9
	Thymus	0.7±0.3	0.6±0.2

Data are presented as mean ± S.D. (*n* = 9) and analyzed via a one-way ANOVA test. ^#^ *p* < 0.05 vs. vehicle-treated mice.

Compared with vehicle-treated *Apc* ^Min/+^ mice, CTP-treated mice showed an increase in the numbers of WBC, LYM, MON, GRA, and PLT in the peripheral blood (Supplementary Table S1). CD3e^−^NK1.1^+^ and CD3e^+^CD28^+^ represent NK cells and activated T cells, respectively, which reflect the levels of cytotoxic immune response (Yan et al., 2018). CTP treatment significantly increased the circulating populations of CD3e^−^NK1.1^+^ cells from 0.56% to 1.20% (*p* < 0.001) (Figure 1C) and CD3e^+^CD28^+^ cells from 2.50% to 4.61% (*p* < 0.01) (Figure 1D) in *Apc* ^Min/+^ mice, as analyzed by flow cytometry. These results showed that CTP suppressed CRC growth primarily by improving the cytotoxic immune response in *Apc* ^Min/+^ mice.

### 3.2 CTP Regulated the Intestinal Microbiota in *Apc* ^Min/+^ Mice

**FIGURE 2:**
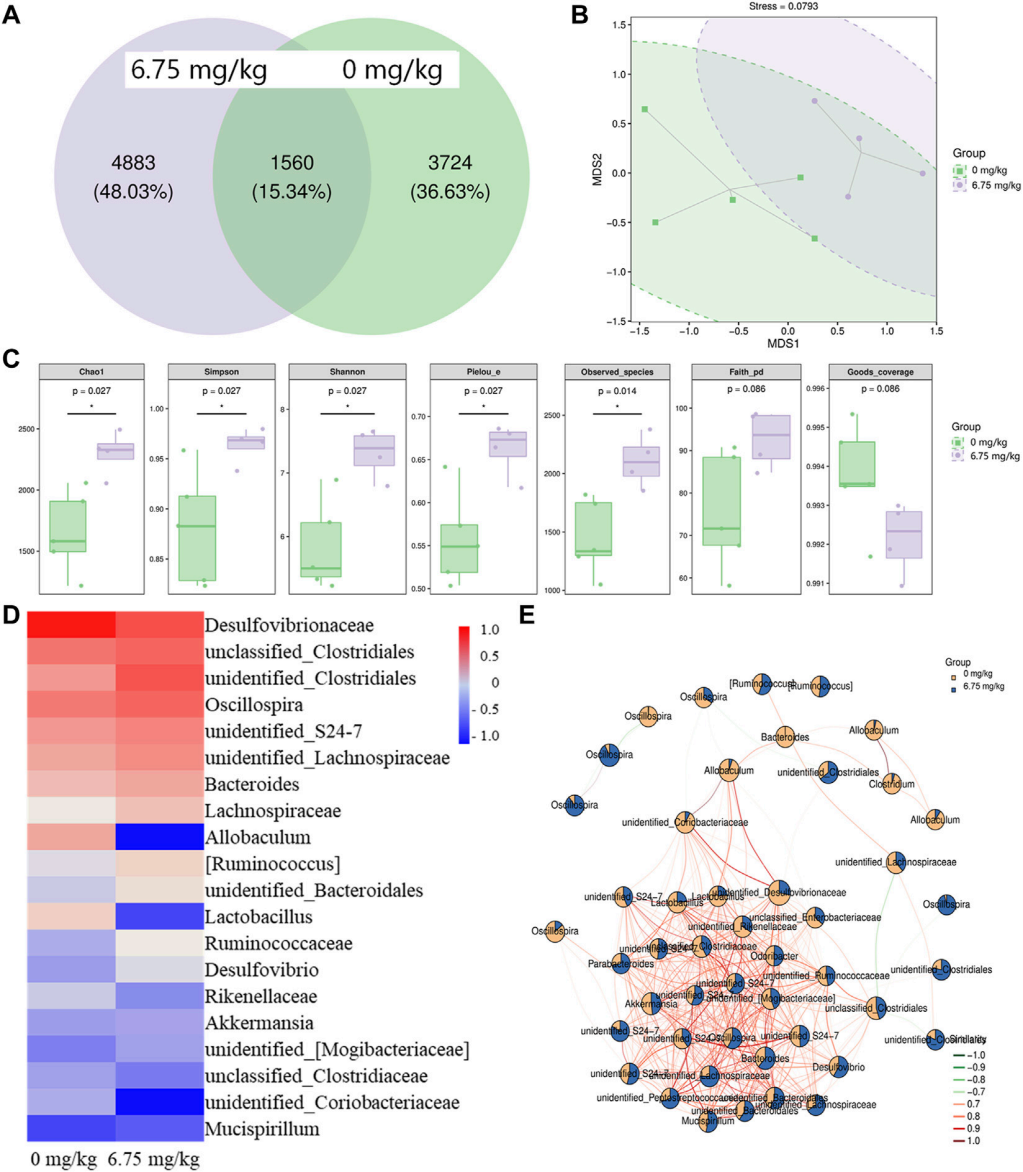
Effect of CTP on the intestinal microbiota of *Apc* ^Min/+^ mice (*n* = 5 for the vehicle-treated group and *n* = 4 for the CTP-treated group). **(A)** Wayne diagram of ASVs in the mice intestinal microbiota. Each circle represents a group, the overlap area indicates the common ASVs number among the two groups, and the number of each block indicates the number of ASVs contained in each block. **(B)** Two-dimensional unweighted UniFrac-based NMDS ordination of the mice intestinal microbiota. The ellipse circles the 95% confidence interval **(C)** Grouping box plot of Alpha diversity index includes Chao1, Simpson, Shannon, Pielou_e, Observed_species, Faith_pd, and Goods_coverage of the mice intestinal microbiota **(D)** Heat map of the top 20 dominant species composition at the genus level and **(E)** the seed network map of the top 50 dominant ASVs with grouping abundance pie chart of the mice intestinal microbiota.

Intestinal microbiota can influence the occurrence and development of CRC (Lenoir et al., 2016; Yamaoka et al., 2018). The Venn result showed that the same number of amplicon sequence variants (ASVs) between the vehicle-treated and CTP-treated *Apc* ^Min/+^ mice was 1,560, proving that CTP could alter the microbiota composition of the cecal contents (Figure 2A). The Krona species composition map intuitively showed the differences in each level between groups (Supplementary Figure S2). The UniFrac NMDS result failed to distinguish between the two tested groups (Figure 2B). Compared with vehicle-treated *Apc* ^Min/+^ mice, in CTP-treated mice, there was a significant increase in five indices (Chao1, Observed species, Simpson, Shannon, and Pielou’s e) (*p* < 0.05) (Figure 2C); consequently, this delineated the regulation of the richness, diversity, and evenness of the intestinal microbiota by CTP. Based on the abundance, CTP caused over a 50% increment in the levels of *unidentified_Clostridiales*, Lachnospiraceae *[Ruminococcus]*, and over a 50% reduction in the levels of Desulfovibrionaceae, *Allobaculum*, *Lactobacillus*, *unidentified_*Coriobacteriaceae on the genus level heat map (Figure 2D). The seed network map of the top 50 dominant ASVs showed that ASVs with complex correlations between nodes were weakly affected by CTP, and ASVs with simple correlations between nodes changed more significantly (Figure 2E). Therefore, CTP may only affect the abundance of specific functional microorganisms.

A linear discriminant analysis effect size (LEfSe) analysis was used to analyze the different biomarkers at all classification levels of the cecal contents (Supplementary Table S2). There were 15 significantly different nodes between vehicle-treated and CTP-treated mice (*p* < 0.05, LDA>2) (Table 2). Erysipelotrichi (class level), Coriobacteriaeae (family level), Desulfovibrionales (order level), and part subordinate nodes demonstrated a high abundance in the cecal contents of vehicle-treated *Apc* ^Min/+^ mice, which decreased significantly after CTP treatment. Cyanobacteria (phylum level), Clostridia (class level), Prevotellaceae (family level), and part subordinate nodes demonstrated a high abundance in the cecal contents of the *Apc* ^Min/+^ mice treated with 6.75 mg/kg CTP (*p* < 0.05, LDA >2) (Table 2).

**TABLE 2:** Dominant nodes based on the LEfSe analysis of intestinal microbiota in *Apc* ^Min/+^ mice.

Group	Taxa	Abundance (log_10_)	LDA score
0 mg/kg	Bacteria.firmicutes.erysipelotrichi	4.681^*^	4.333
	Bacteria.firmicutes.erysipelotrichi.erysipelotrichales	4.694^*^	4.339
	Bacteria.firmicutes.frysipelotrichi.erysipelotrichales.erysipelotrichaceae.*allobaculum*	4.694^*^	4.336
	Bacteria.actinobacteria.coriobacteriia.coriobacteriales.coriobacteriaceae	4.114^*^	3.876
	Bacteria.proteobacteria.deltaproteobacteria.desulfovibrionales	5.545^*^	4.859
	Bacteria.proteobacteria.deltaproteobacteria.desulfovibrionales.desulfovibrionaceae	5.531^*^	4.886
6.75 mg/kg	Bacteria.cyanobacteria	2.968^#^	4.074
	Bacteria.cyanobacteria.4C0d_2.YS2	2.968^#^	4.059
	Bacteria.cyanobacteria.4C0d_2	2.968^#^	4.063
	Bacteria.firmicutes.clostridia	5.773^#^	5.017
	Bacteria.firmicutes.clostridia.clostridiales.lachnospiraceae.*coprococcus*	4.011^#^	3.979
	Bacteria.firmicutes.clostridia.clostridiales.lachnospiraceae._*ruminococcus*_	4.425^#^	3.856
	Bacteria.firmicutes.clostridia.clostridiales.clostridiaceae.*clostridium*	2.704^#^	4.646
	Bacteria.bacteroidetes.bacteroidia.bacteroidales.prevotellaceae	2.408^#^	4.169
	Bacteria.bacteroidetes.bacteroidia.bacteroidales._paraprevotellaceae_.*_prevotella_*	4.020^##^	3.790

Data are presented as mean and analyzed *via* a Wilcoxon test (*n* = 5 for the vehicle-treated group and *n* = 4 for the CTP-treated group). The advantaged group, logarithm abundance of dominant nodes, and LDA scores are provided. ^*^ *p* < 0.05 vs. CTP-treated mice; ^#^ *p* < 0.05 and ^##^ *p* < 0.01 vs. vehicle-treated mice.

### 3.3 Effect of CTP on Cytokines Levels of Serum and Colorectum in *Apc* ^Min/+^ Mice

**FIGURE 3:**
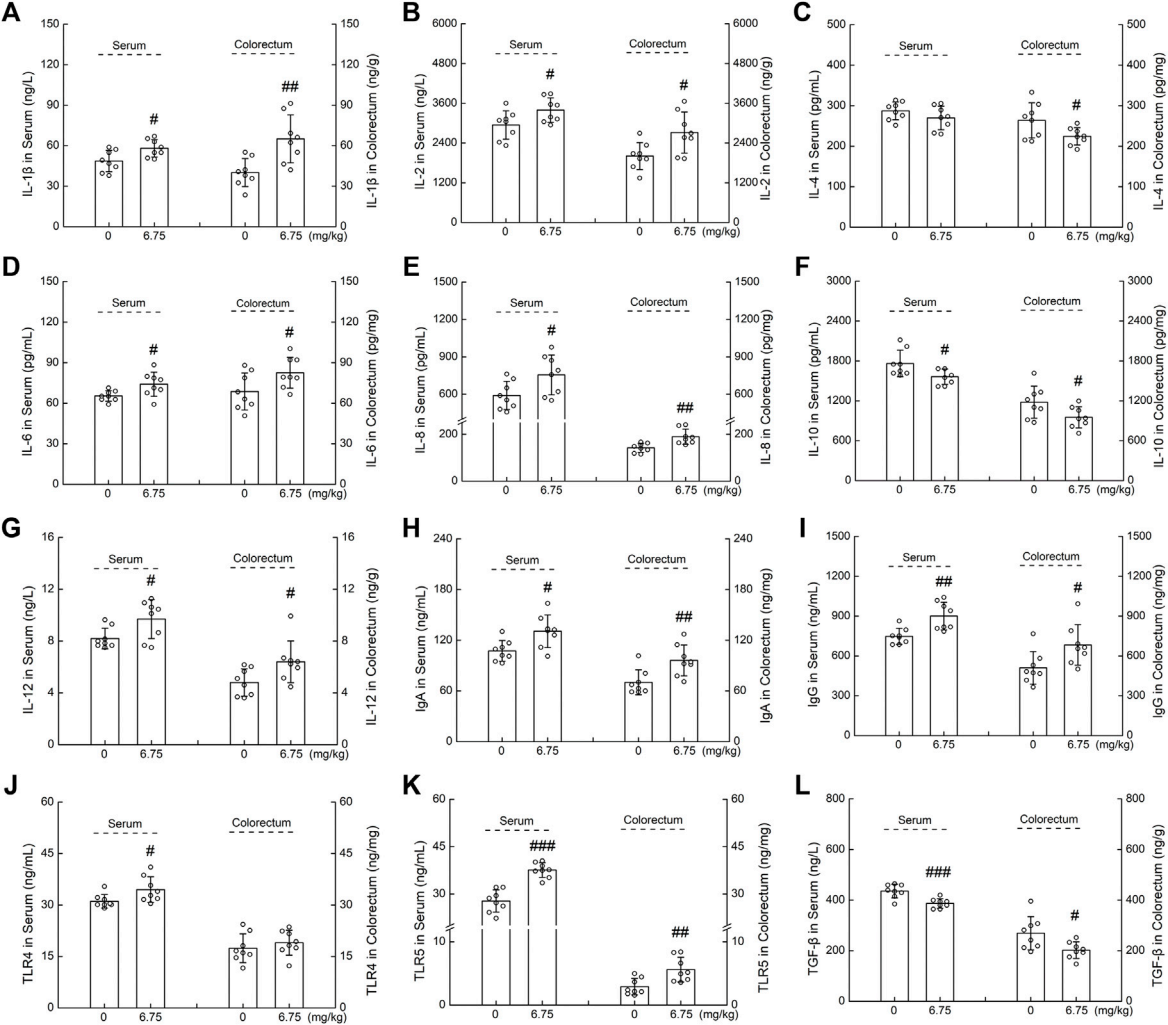
The effects of CTP on serum and colorectum cytokines of ApcMin/+ mice, including **(A)** IL-1β, **(B)** IL-2, **(C)** IL-4, **(D)** IL-6, **(E)** IL-8, **(F)** IL-10, **(G)** IL-12, **(H)** IgA, **(I)** IgG, **(J)** TLR4, **(K)** TLR5, and **(L)** TGF-β. Data are presented as the mean ± S.D. and analyzed via a one-way ANOVA test (*n* = 8). ^#^ *p* < 0.05, ^##^ *p* < 0.01, and ^###^ *p* < 0.001 vs. vehicle-treated mice.

The levels of immune-related cytokines were analyzed to evaluate the immunomodulatory effect of CTP in *Apc* ^Min/+^ mice. Compared with the vehicle-treated *Apc* ^Min/+^ mice, CTP treatment significantly enhanced the serum levels of IL-1β (19.50%) (*p* < 0.05) (Figure 3A), IL-2 (15.19%) (*p* < 0.05) (Figure 3B), IL-6 (13.29%) (*p* < 0.05) (Figure 3D), IL-8 (27.95%) (*p* < 0.05) (Figure 3E), IL-12 (17.47%) (*p* < 0.05) (Figure 3G), IgA (21.70%) (*p* < 0.05) (Figure 3H), IgG (20.42%) (*p* < 0.01) (Figure 3I), TLR4 (10.82%) (*p* < 0.05) (Figure 3J), TLR5 (35.22%) (*p* < 0.001) (Figure 3K), and enhanced colorectum levels of IL-1β (62.29%) (*p* < 0.01) (Figure 3A), IL-2 (35.30%) (*p* < 0.05) (Figure 3B), IL-6 (20.07%) (*p* < 0.05) (Figure 3D), IL-8 (33.24%) (*p* < 0.01) (Figure 3E), IL-12 (32.48%) (*p* < 0.05) (Figure 3G), IgA (37.18%) (*p* < 0.01) (Figure 3H), IgG (33.82%) (*p* < 0.05) (Figure 3I), and TLR5 (94.21%) (*p* < 0.01) (Figure 3K). Moreover, CTP reduced the serum levels of IL-10 (11.35%) (*p* < 0.05) (Figure 3F), and TGF-β (11.19%) (*p* < 0.001) (Figure. 3L), and reduced the colon levels of IL-4 (15.01%) (*p* < 0.05) (Figure 3C), IL-10 (19.28%) (*p* < 0.05) (Figure 3F), and TGF-β (24.84%) (*p* < 0.05) (Figure 3L) in *Apc* ^Min/+^ mice. CTP induced an immunomodulatory effect in *Apc* ^Min/+^ mice.

### 3.4 CTP Regulated the Immune Function *via* IL-2-Associated Signaling Pathway in *Apc* ^Min/+^ Mice

**FIGURE 4:**
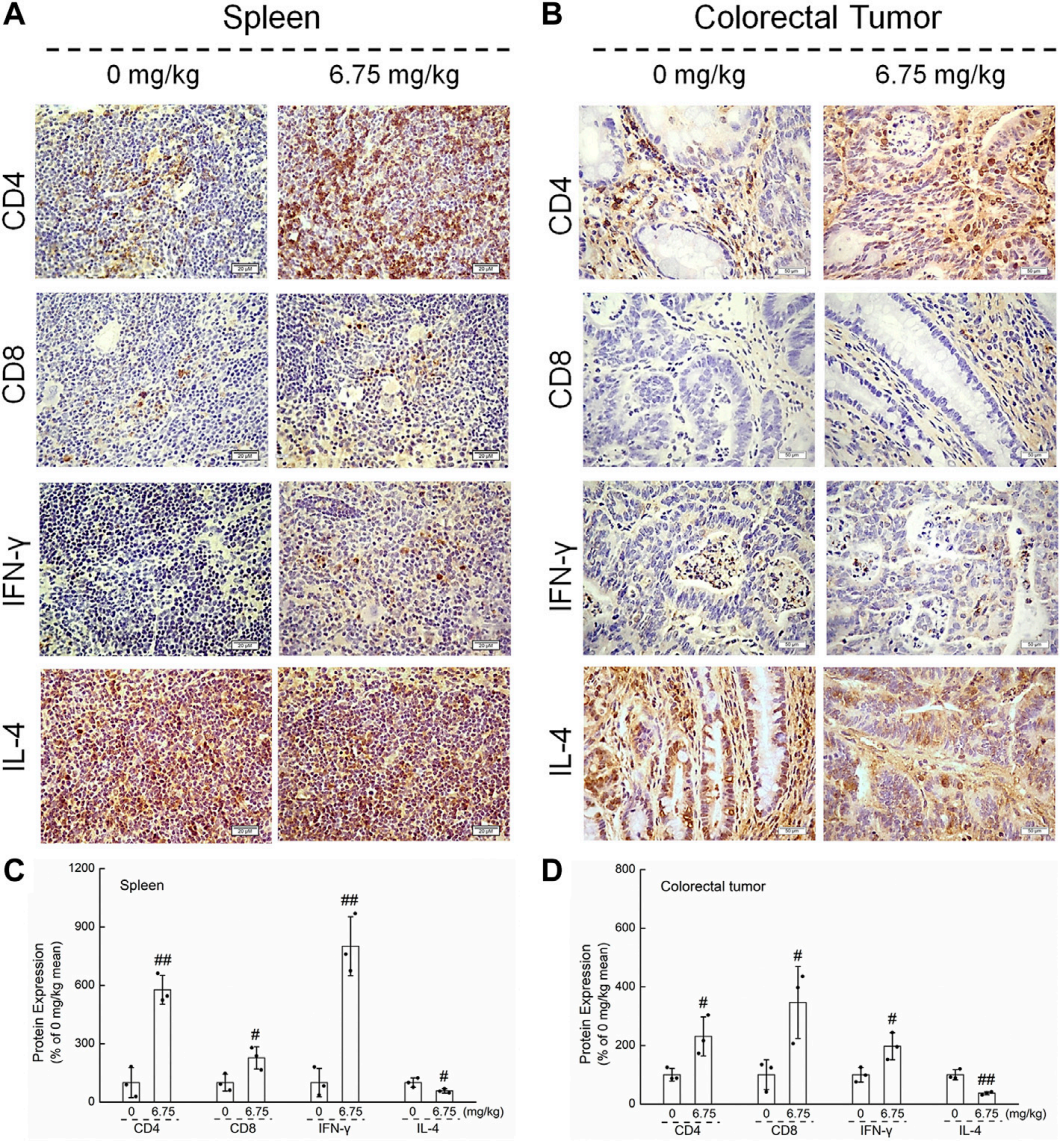
Effects of CTP on CD4, CD8, IFN-γ, and IL-4 of the spleen and colorectal tumor in *Apc* ^Min/+^ mice. CTP enhanced the levels of CD4, CD8, and IFN-γ, and suppressed the levels of IL-4 in **(A)** the spleen (400 ×, scale bar: 20 μm) and **(B)** colorectal tumor (200 ×, scale bar: 50 μm) analyzed by immunohistochemistry. The pixel density for the semi-quantitative densitometric analysis of protein expressions in **(C)** the spleen and **(D)** colorectal tumors were quantified. Data are presented as mean ± S.D. and analyzed *via* a one-way ANOVA test (*n* = 3). ^#^ *p* < 0.05 and ^##^ *p* < 0.01 vs. vehicle-treated mice.

Immunohistochemistry and Western blot analysis were used to explore the relationship between the suppressive effect of CTP on CRC and immune regulation in *Apc* ^Min/+^ mice. CTP significantly increased the levels of CD4 and CD8, enhanced the expression of IFN-γ, and suppressed the levels of IL-4 in the spleen (*p* < 0.05) (Figures 4A,C) and colorectal tumors (*p* < 0.05) (Figures 4B,D) of *Apc* ^Min/+^ mice, suggesting the increased number of CD8^+^ and CD4^+^ cells and the increased proportion of helper T lymphocytes (Th)1/Th2 in CTP-treated *Apc* ^Min/+^ mice.

**FIGURE 5:**
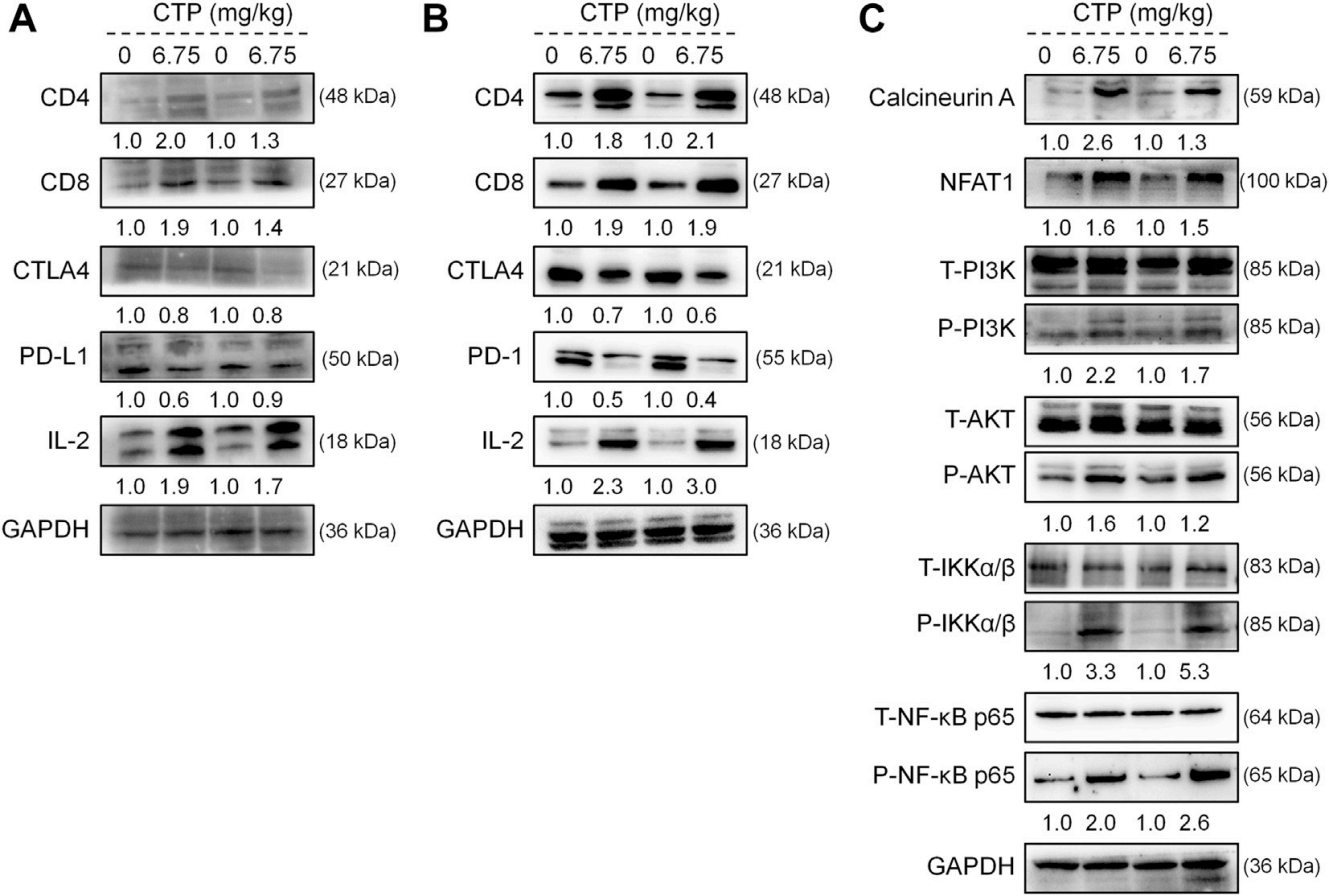
CTP regulated the expression of proteins associated with immunity in the spleen and colorectal tumors of *Apc* ^Min/+^ mice. CTP regulated the expression levels of CD4, CD8, CTLA4, PD-L1, and IL-2 in **(A)** the colorectal tumor and **(B)** spleen of *Apc* ^Min/+^ mice **(C)** CTP regulated the expression levels of Calcineurin A, NFAT1, P-PI3K, P-AKT, P-IKKα/β, and P-NF-κB p65 in the spleen of *Apc* ^Min/+^ mice. Quantification data were normalized by GAPDH and their corresponding total proteins were reported as fold change with respect to the expression data from the corresponding vehicle-treated mice (*n* = 4).

According to the results of the Western blot, CTP significantly increased the levels of CD4, CD8, and IL-2, and reduced the levels of CTLA4 and PD-L1 in the tumor (Figure 5A), and CTLA4 and PD-1 in the spleen (Figure 5B) of *Apc* ^Min/+^ mice; this underscored the pivotal role of IL-2 in the immune response. Furthermore, CTP significantly increased the expression levels of calcineurin A, NFAT1, P-PI3K, P-AKT, P-IKKα/β, and P-NF-κB p65 in the spleen of *Apc* ^Min/+^ mice (Figure 5C), indicating the activation of the NFAT1 and NF-κB p65 pathways.

## 4 Discussion

In the present study, the anti-CRC effect of CTP was systematically confirmed in the *Apc* ^Min/+^ mice model, which was achieved by regulating the composition of the intestinal microbiota and enhancing the immune cytolytic activity of both NK cells and cytotoxic T cells.

CTP effectively limited the development of CRC and modulated the abundance of the intestinal microbiota in *Apc* ^Min/+^ mice. The total number of microbes may reach 100 trillion in the adult human intestine (Barengolts, 2013), which has been shown to play specific roles in various diseases. Accordingly, secondary metabolites of *Clostridia* can effectively regulate immunity and limit chronic inflammation, thereby impeding the occurrence and development of CRC (Reichardt et al., 2014; Zhang Z et al., 2019). *Allobaculum* is considered an indicator of defective immune responses in CD45-deficient mice (Dimitriu et al., 2013). Desulfovibrionales is an important source of cytotoxic compounds, such as toxins and hydrogen sulfide in the intestine, and causes various diseases (Loubinoux et al., 2000; Sawin et al., 2015). Furthermore, its reduced abundance helps to inhibit inflammation-associated CRC (Zhang H et al., 2019). CTP significantly increased the abundance of *Clostridia*, and reduced the abundance of *Allobaculum* and Desulfovibrionales, suggesting that the anti-CRC effect of CTP is partially related to its modulation of the intestinal microbiota, which may influence immune regulation.

The aggregation of T cells in the tumor tissue reflects an improvement in the prognosis of patients with CRC (Pages et al., 2009; Tosolini et al., 2011). CD8^+^ cytotoxic T cells are the primary participants driving the adaptive immune response against cancer and executing a tumor-specific immune response; therefore, CD8^+^ cytotoxic T cells are the primary endpoints of immunotherapy (Spitzer et al., 2017; Ostroumov et al., 2018). On the one hand, activated CD4^+^ T cells secrete various cytokines, such as IFN- γ and IL-2 (Kershaw et al., 2013; Farhood et al., 2019); on the other hand, CD4^+^ T cells promote the costimulatory molecules and cytokine expressions of dendritic cells, all of which contribute to CD8^+^ T-cell activation (Gottschalk et al., 2015). Importantly, CD4^+^ Th1 cells (expressing IL-2 and IFN-γ) promote the cytotoxic immune response, and CD4^+^ Th2 cells (expressing IL-4 and IL-10) promote the humoral immune response and limit the cytotoxic immune response (Jankovic et al., 2001). CD28, a co-stimulatory molecule required for T-cell activation, is another factor involved in CD8^+^ cytotoxic T-cell activation (Schwartz, 1992). In the present study, CTP enhanced the levels of IFN-γ and suppressed the levels of IL-4, suggesting an increase in the Th1/Th2 ratio (Cortés et al., 2017; Yang and Zhou, 2019). CTP enhances the levels of TLR4 and TLR5 in *Apc* ^Min/+^ mice, indicating an enhanced tumor killing response mediated by T cells (Brackett et al., 2016; Farrokhi et al., 2019). CTP not only enhanced the activation of T cells (CD3e^+^CD28^+^) but also increased the number of NK cells (CD3e^−^NK1.1^+^) in the peripheral blood of *Apc* ^Min/+^ mice. As non-specific killer cells, NK cells can promote specific immune responses or directly kill target cells via antibody-dependent pathways. A decrease in the percentage of NK cells in the peripheral blood usually leads to a high incidence of tumors (Vivier et al., 2012; Panda et al., 2017). In CTP-treated *Apc* ^Min/+^ mice, the enhanced IL-2 and IL-12 levels were confirmed, indicating the promotion of T-cell and NK-cell activation (Carballido et al., 2012; Yan et al., 2018), and promotion of Th1 cells’ differentiation (Yan et al., 2018). The increase in IgG and reduction in TGF-β after CTP administration further confirmed the activation of NK cells in *Apc* ^Min/+^ mice (Viel et al., 2016; Bournazos and Ravetch, 2017), which is also consistent with the findings of our previous study (Li et al., 2020). The inhibitory effect of CTP on CRC is partially linked to the promotion of the tumor immune killing response in *Apc* ^Min/+^ mice, especially the activation of T cells and NK cells.

CTP reduces the levels of PD-1, PD-L1, and CTLA4 in *Apc* ^Min/+^ mice, which have been confirmed to regulate the immune reactivity of tumor cells (Sansom, 2000; de Vries et al., 2016), and antibodies against PD-1 and PD-L1 have been successfully used for tumor therapy (Sunshine and Taube, 2015). The blockade of CTLA-4 and PD-1/PD-L1 promotes the proliferation of CD8^+^ T cells and the expression of IL-2 (Spranger et al., 2014), and, the increase of IL-2 expression reverses the T-cell inhibition caused by PD-1 and its ligands (Carter et al., 2002). The activation of NF-κB signaling is a key step for inducing IL-2 gene transcription in T cells. Moreover, the co-stimulatory signaling from NFAT1 and the calcium influx of T cells results in increased IL-2 and IL-2 receptors (Clavijo and Frauwirth, 2012; Lee et al., 2018). CTP strongly enhanced the activation of NFAT1 and NF-κB p65 signaling in *Apc* ^Min/+^ mice. The activation of NFAT1 and NF-κB signaling promotes the activation and differentiation of CD8^+^ T cells and helps in maintaining the activity of CD8^+^ T cells (Clavijo and Frauwirth, 2012; Lee et al., 2018). The inhibitory effect of CTP on CRC is due to promoting the immune function related to the IL-2-associated signaling pathway.

The present study has certain limitations. Accordingly, thymosin promotes T-cell maturation by increasing IL-7 synthesis (Knutsen et al., 1999), and thymosin alpha 1 increases major histocompatibility complex class-1, antagonized dexamethasone-induced apoptosis of CD4^+^CD8^+^ cells (Baumann et al., 2000). Although our present results confirmed the inhibition effect on CRC of CTP associated with T cells, the detailed mechanism of its differentiation remains to be investigated in a future study. Second, as a clinical drug, the anti-CRC effect of CTP in *Apc* ^Min/+^ mice was confirmed, which requires follow-up clinical verification.

Altogether, CTP can inhibit the development of CRC in *Apc* ^Min/+^ mice, which is related to intestinal microbiota changes and immune response regulation. Our data provide a reference for CTP as a therapeutic drug or combination drug used in clinical CRC treatment.

## Data Availability

The datasets presented in this study can be found in online repositories. The names of the repository/repositories and accession number(s) can be found in the article/Supplementary Material.
