# Supplementary material for: Calf Thymus Polypeptide Restrains the Growth of Colorectal Tumor via Regulating the Intestinal Microbiota-Mediated Immune Function
Source: Front Pharmacol. 2022 May 19;13:898906. doi: 10.3389/fphar.2022.898906 (PMC9160181; doi:10.3389/fphar.2022.898906)
Supplement: Supplementary file 1 [file DataSheet1.docx]

Supplementary Material

**Supplementary Table S1** The effect of CTP on peripheral blood of *Apc*^Min/+^ mice.

|  | 0 mg/kg CTP | 6.75 mg/kg CTP |
| --- | --- | --- |
| WBC (*10^9^/L) | 63.4±31.6 | 83.1±23.8 |
| LYM (*10^9^/L) | 38.5±17.9 | 48.7±11.7 |
| MON (*10^9^/L) | 11.5±7.9 | 15.9±10.6 |
| GRA (*10^9^/L) | 13.3±3.1 | 18.4±1.5 |
| RBC (*10^12^/L) | 3.9±1 | 3.4±1.8 |
| MCV (fL) | 67±2.9 | 63.3±12 |
| PLT (*10^9^/L) | 1026.3±312.2 | 1382.5±760.1 |
| MPV (fL) | 11.6±0.7 | 11.7±1.3 |

Data are presented as the mean ± SD (n = 3).

**Supplementary Table S2** The LEfSe analysis result on intestinal microbiota of *Apc*^Min/+^ mice.

|  | Taxa | Abundace（log10） | LDA score | Group |
| --- | --- | --- | --- | --- |
| 1 | Bacteria.Acidobacteria | 0.827 |  |  |
| 2 | Bacteria.Acidobacteria.Acidobacteria_6 | 0.827 |  |  |
| 3 | Bacteria.Acidobacteria.Acidobacteria_6.iii1_15 | 0.827 |  |  |
| 4 | Bacteria.Acidobacteria.Acidobacteria_6.iii1_15.mb2424 | 0.827 |  |  |
| 5 | Bacteria.Actinobacteria | 4.192 |  |  |
| 6 | Bacteria.Actinobacteria.Actinobacteria | 3.275 |  |  |
| 7 | Bacteria.Actinobacteria.Actinobacteria.Actinomycetales | 1.807 |  |  |
| 8 | Bacteria.Actinobacteria.Actinobacteria.Actinomycetales.Corynebacteriaceae | 1.767 |  |  |
| 9 | Bacteria.Actinobacteria.Actinobacteria.Actinomycetales.Corynebacteriaceae.Corynebacterium | 1.767 |  |  |
| 10 | Bacteria.Actinobacteria.Actinobacteria.Actinomycetales.Micrococcaceae | 0.746 |  |  |
| 11 | Bacteria.Actinobacteria.Actinobacteria.Actinomycetales.Micrococcaceae.Rothia | 0.746 |  |  |
| 12 | Bacteria.Actinobacteria.Actinobacteria.Actinomycetales.Streptomycetaceae | 1.304 |  |  |
| 13 | Bacteria.Actinobacteria.Actinobacteria.Actinomycetales.Streptomycetaceae.Streptomyces | 1.304 |  |  |
| 14 | Bacteria.Actinobacteria.Actinobacteria.Bifidobacteriales | 3.26 |  |  |
| 15 | Bacteria.Actinobacteria.Actinobacteria.Bifidobacteriales.Bifidobacteriaceae | 0.949 |  |  |
| 16 | Bacteria.Actinobacteria.Actinobacteria.Bifidobacteriales.Bifidobacteriaceae.Bifidobacterium | 3.258 |  |  |
| 17 | Bacteria.Actinobacteria.Coriobacteriia | 4.136 |  |  |
| 18 | Bacteria.Actinobacteria.Coriobacteriia.Coriobacteriales | 4.136 |  |  |
| **19** | **Bacteria.Actinobacteria.Coriobacteriia.Coriobacteriales.Coriobacteriaceae** | **4.114** | **3.87615** | **0 mg/kg CTP** |
| 20 | Bacteria.Actinobacteria.Coriobacteriia.Coriobacteriales.Coriobacteriaceae.Adlercreutzia | 2.908 |  |  |
| 21 | Bacteria.Actinobacteria.Coriobacteriia.Coriobacteriales.Coriobacteriaceae.Olsenella | 1.767 |  |  |
| 22 | Bacteria.Bacteroidetes | 5.265 |  |  |
| 23 | Bacteria.Bacteroidetes.Bacteroidia | 5.265 |  |  |
| 24 | Bacteria.Bacteroidetes.Bacteroidia.Bacteroidales | 4.362 |  |  |
| 25 | Bacteria.Bacteroidetes.Bacteroidia.Bacteroidales._Odoribacteraceae_ | 3.78 |  |  |
| 26 | Bacteria.Bacteroidetes.Bacteroidia.Bacteroidales._Odoribacteraceae_.Odoribacter | 3.78 |  |  |
| 27 | Bacteria.Bacteroidetes.Bacteroidia.Bacteroidales._Paraprevotellaceae_ | 0.918 |  |  |
| **28** | **Bacteria.Bacteroidetes.Bacteroidia.Bacteroidales._Paraprevotellaceae_._Prevotella_** | **4.02** | **3.79031** | **6.75 mg/kg CTP** |
| 29 | Bacteria.Bacteroidetes.Bacteroidia.Bacteroidales.Bacteroidaceae | 4.689 |  |  |
| 30 | Bacteria.Bacteroidetes.Bacteroidia.Bacteroidales.Bacteroidaceae.Bacteroides | 4.689 |  |  |
| 31 | Bacteria.Bacteroidetes.Bacteroidia.Bacteroidales.Porphyromonadaceae | 3.93 |  |  |
| 32 | Bacteria.Bacteroidetes.Bacteroidia.Bacteroidales.Porphyromonadaceae.Parabacteroides | 3.93 |  |  |
| **33** | **Bacteria.Bacteroidetes.Bacteroidia.Bacteroidales.Prevotellaceae** | **2.408** | **4.16998** | **6.75 mg/kg CTP** |
| 34 | Bacteria.Bacteroidetes.Bacteroidia.Bacteroidales.Prevotellaceae.Prevotella | 1.301 |  |  |
| 35 | Bacteria.Bacteroidetes.Bacteroidia.Bacteroidales.Rikenellaceae | 4.187 |  |  |
| 36 | Bacteria.Bacteroidetes.Bacteroidia.Bacteroidales.Rikenellaceae.Alistipes | 2.716 |  |  |
| 37 | Bacteria.Bacteroidetes.Bacteroidia.Bacteroidales.S24_7 | 4.881 |  |  |
| 38 | Bacteria.Bacteroidetes.Flavobacteriia | 0.648 |  |  |
| 39 | Bacteria.Bacteroidetes.Flavobacteriia.Flavobacteriales | 0.648 |  |  |
| 40 | Bacteria.Bacteroidetes.Flavobacteriia.Flavobacteriales.Flavobacteriaceae | 0.648 |  |  |
| 41 | Bacteria.Bacteroidetes.Flavobacteriia.Flavobacteriales.Flavobacteriaceae.Flavobacterium | 0.648 |  |  |
| 42 | **Bacteria.Cyanobacteria** | **2.968** | **4.07472** | **6.75 mg/kg CTP** |
| 43 | **Bacteria.Cyanobacteria.4C0d_2** | **2.968** | **4.06384** | **6.75 mg/kg CTP** |
| 44 | **Bacteria.Cyanobacteria.4C0d_2.YS2** | **2.968** | **4.05980** | **6.75 mg/kg CTP** |
| 45 | Bacteria.Deferribacteres | 3.89 |  |  |
| 46 | Bacteria.Deferribacteres.Deferribacteres | 3.89 |  |  |
| 47 | Bacteria.Deferribacteres.Deferribacteres.Deferribacterales | 3.89 |  |  |
| 48 | Bacteria.Deferribacteres.Deferribacteres.Deferribacterales.Deferribacteraceae | 3.89 |  |  |
| 49 | Bacteria.Deferribacteres.Deferribacteres.Deferribacterales.Deferribacteraceae.Mucispirillum | 3.89 |  |  |
| 50 | Bacteria.Firmicutes | 2.38 |  |  |
| 51 | Bacteria.Firmicutes.Bacilli | 4.446 |  |  |
| 52 | Bacteria.Firmicutes.Bacilli.Bacillales | 0.766 |  |  |
| 53 | Bacteria.Firmicutes.Bacilli.Bacillales.Staphylococcaceae | 0.666 |  |  |
| 54 | Bacteria.Firmicutes.Bacilli.Bacillales.Staphylococcaceae.Staphylococcus | 0.766 |  |  |
| 55 | Bacteria.Firmicutes.Bacilli.Lactobacillales | 1.223 |  |  |
| 56 | Bacteria.Firmicutes.Bacilli.Lactobacillales.Enterococcaceae | 0.646 |  |  |
| 57 | Bacteria.Firmicutes.Bacilli.Lactobacillales.Enterococcaceae.Enterococcus | 1.499 |  |  |
| 58 | Bacteria.Firmicutes.Bacilli.Lactobacillales.Lactobacillaceae | 1.137 |  |  |
| 59 | Bacteria.Firmicutes.Bacilli.Lactobacillales.Lactobacillaceae.Lactobacillus | 4.44 |  |  |
| 60 | Bacteria.Firmicutes.Bacilli.Lactobacillales.Leuconostocaceae | 1.468 |  |  |
| 61 | Bacteria.Firmicutes.Bacilli.Lactobacillales.Leuconostocaceae.Leuconostoc | 1.468 |  |  |
| 62 | Bacteria.Firmicutes.Bacilli.Lactobacillales.Streptococcaceae | 0.648 |  |  |
| 63 | Bacteria.Firmicutes.Bacilli.Lactobacillales.Streptococcaceae.Lactococcus | 1.953 |  |  |
| 64 | Bacteria.Firmicutes.Bacilli.Lactobacillales.Streptococcaceae.Streptococcus | 2.398 |  |  |
| 65 | Bacteria.Firmicutes.Bacilli.Turicibacterales | 1.694 |  |  |
| 66 | Bacteria.Firmicutes.Bacilli.Turicibacterales.Turicibacteraceae | 1.694 |  |  |
| 67 | Bacteria.Firmicutes.Bacilli.Turicibacterales.Turicibacteraceae.Turicibacter | 1.694 |  |  |
| **68** | **Bacteria.Firmicutes.Clostridia** | **5.773** | **5.01797** | **6.75 mg/kg CTP** |
| 69 | Bacteria.Firmicutes.Clostridia.Clostridiales | 5.421 |  |  |
| 70 | Bacteria.Firmicutes.Clostridia.Clostridiales._Mogibacteriaceae_ | 4.074 |  |  |
| 71 | Bacteria.Firmicutes.Clostridia.Clostridiales._Mogibacteriaceae_.Mogibacterium | 0.717 |  |  |
| 72 | Bacteria.Firmicutes.Clostridia.Clostridiales._Tissierellaceae_ | 1.426 |  |  |
| 73 | Bacteria.Firmicutes.Clostridia.Clostridiales._Tissierellaceae_.Anaerococcus | 0.949 |  |  |
| 74 | Bacteria.Firmicutes.Clostridia.Clostridiales._Tissierellaceae_.Gallicola | 0.949 |  |  |
| 75 | Bacteria.Firmicutes.Clostridia.Clostridiales._Tissierellaceae_.GW_34 | 0.648 |  |  |
| 76 | Bacteria.Firmicutes.Clostridia.Clostridiales._Tissierellaceae_.Peptoniphilus | 0.648 |  |  |
| 77 | Bacteria.Firmicutes.Clostridia.Clostridiales.Christensenellaceae | 3.115 |  |  |
| 78 | Bacteria.Firmicutes.Clostridia.Clostridiales.Clostridiaceae | 4.073 |  |  |
| 79 | Bacteria.Firmicutes.Clostridia.Clostridiales.Clostridiaceae.02d06 | 2.5 |  |  |
| **80** | **Bacteria.Firmicutes.Clostridia.Clostridiales.Clostridiaceae.Clostridium** | **2.704** | **4.64634** | **6.75 mg/kg CTP** |
| 81 | Bacteria.Firmicutes.Clostridia.Clostridiales.Clostridiaceae.SMB53 | 1.994 |  |  |
| 82 | Bacteria.Firmicutes.Clostridia.Clostridiales.Dehalobacteriaceae | 3.436 |  |  |
| 83 | Bacteria.Firmicutes.Clostridia.Clostridiales.Dehalobacteriaceae.Dehalobacterium | 3.436 |  |  |
| 84 | Bacteria.Firmicutes.Clostridia.Clostridiales.Eubacteriaceae | 1.243 |  |  |
| 85 | Bacteria.Firmicutes.Clostridia.Clostridiales.Eubacteriaceae.Anaerofustis | 1.243 |  |  |
| 86 | Bacteria.Firmicutes.Clostridia.Clostridiales.Lachnospiraceae | 5.011 |  |  |
| **87** | **Bacteria.Firmicutes.Clostridia.Clostridiales.Lachnospiraceae._Ruminococcus_** | **4.425** | **3.85634** | **6.75 mg/kg CTP** |
| 88 | Bacteria.Firmicutes.Clostridia.Clostridiales.Lachnospiraceae.Clostridium | 3.836 |  |  |
| **89** | **Bacteria.Firmicutes.Clostridia.Clostridiales.Lachnospiraceae.Coprococcus** | **4.011** | **3.97987** | **6.75 mg/kg CTP** |
| 90 | Bacteria.Firmicutes.Clostridia.Clostridiales.Lachnospiraceae.Dorea | 3.15 |  |  |
| 91 | Bacteria.Firmicutes.Clostridia.Clostridiales.Lachnospiraceae.Roseburia | 2.751 |  |  |
| 92 | Bacteria.Firmicutes.Clostridia.Clostridiales.Peptococcaceae | 2.924 |  |  |
| 93 | Bacteria.Firmicutes.Clostridia.Clostridiales.Peptostreptococcaceae | 3.97 |  |  |
| 94 | Bacteria.Firmicutes.Clostridia.Clostridiales.Peptostreptococcaceae.Clostridium | 1.655 |  |  |
| 95 | Bacteria.Firmicutes.Clostridia.Clostridiales.Ruminococcaceae | 4.406 |  |  |
| 96 | Bacteria.Firmicutes.Clostridia.Clostridiales.Ruminococcaceae.Anaerotruncus | 3.541 |  |  |
| 97 | Bacteria.Firmicutes.Clostridia.Clostridiales.Ruminococcaceae.Butyricicoccus | 2.164 |  |  |
| 98 | Bacteria.Firmicutes.Clostridia.Clostridiales.Ruminococcaceae.Clostridium | 1.243 |  |  |
| 99 | Bacteria.Firmicutes.Clostridia.Clostridiales.Ruminococcaceae.Oscillospira | 5.059 |  |  |
| 100 | Bacteria.Firmicutes.Clostridia.Clostridiales.Ruminococcaceae.Ruminococcus | 3.807 |  |  |
| 101 | Bacteria.Firmicutes.Clostridia.Clostridiales.Veillonellaceae | 0.648 |  |  |
| 102 | Bacteria.Firmicutes.Clostridia.SHA_98 | 1.389 |  |  |
| **103** | **Bacteria.Firmicutes.Erysipelotrichi** | **4.694** | **4.33672** | **0 mg/kg CTP** |
| **104** | **Bacteria.Firmicutes.Erysipelotrichi.Erysipelotrichales** | **4.694** | **4.33928** | **0 mg/kg CTP** |
| 105 | Bacteria.Firmicutes.Erysipelotrichi.Erysipelotrichales.Erysipelotrichaceae | 2.563 |  |  |
| **106** | **Bacteria.Firmicutes.Erysipelotrichi.Erysipelotrichales.Erysipelotrichaceae.Allobaculum** | **4.682** | **4.33302** | **0 mg/kg CTP** |
| 107 | Bacteria.Firmicutes.Erysipelotrichi.Erysipelotrichales.Erysipelotrichaceae.Bulleidia | 0.842 |  |  |
| 108 | Bacteria.Firmicutes.Erysipelotrichi.Erysipelotrichales.Erysipelotrichaceae.Clostridium | 3.019 |  |  |
| 109 | Bacteria.Gemmatimonadetes | 0.827 |  |  |
| 110 | Bacteria.Gemmatimonadetes.Gemmatimonadetes | 0.827 |  |  |
| 111 | Bacteria.Proteobacteria | 0.83 |  |  |
| 112 | Bacteria.Proteobacteria.Alphaproteobacteria | 2.144 |  |  |
| 113 | Bacteria.Proteobacteria.Alphaproteobacteria.Caulobacterales | 1.192 |  |  |
| 114 | Bacteria.Proteobacteria.Alphaproteobacteria.Caulobacterales.Caulobacteraceae | 1.192 |  |  |
| 115 | Bacteria.Proteobacteria.Alphaproteobacteria.Caulobacterales.Caulobacteraceae.Caulobacter | 1.192 |  |  |
| 116 | Bacteria.Proteobacteria.Alphaproteobacteria.Rhizobiales | 0.746 |  |  |
| 117 | Bacteria.Proteobacteria.Alphaproteobacteria.Rhizobiales.Beijerinckiaceae | 0.646 |  |  |
| 118 | Bacteria.Proteobacteria.Alphaproteobacteria.Rhizobiales.Beijerinckiaceae.Chelatococcus | 0.646 |  |  |
| 119 | Bacteria.Proteobacteria.Alphaproteobacteria.Rhodospirillales | 0.993 |  |  |
| 120 | Bacteria.Proteobacteria.Alphaproteobacteria.Rhodospirillales.Rhodospirillaceae | 0.993 |  |  |
| 121 | Bacteria.Proteobacteria.Alphaproteobacteria.Rhodospirillales.Rhodospirillaceae.Magnetospirillum | 0.993 |  |  |
| 122 | Bacteria.Proteobacteria.Alphaproteobacteria.Sphingomonadales | 2.017 |  |  |
| 123 | Bacteria.Proteobacteria.Alphaproteobacteria.Sphingomonadales.Sphingomonadaceae | 1.47 |  |  |
| 124 | Bacteria.Proteobacteria.Alphaproteobacteria.Sphingomonadales.Sphingomonadaceae.Sphingomonas | 1.871 |  |  |
| 125 | Bacteria.Proteobacteria.Betaproteobacteria | 1.107 |  |  |
| 126 | Bacteria.Proteobacteria.Betaproteobacteria.Burkholderiales | 2.535 |  |  |
| 127 | Bacteria.Proteobacteria.Betaproteobacteria.Burkholderiales.Burkholderiaceae | 2.398 |  |  |
| 128 | Bacteria.Proteobacteria.Betaproteobacteria.Burkholderiales.Burkholderiaceae.Burkholderia | 2.398 |  |  |
| 129 | Bacteria.Proteobacteria.Betaproteobacteria.Burkholderiales.Comamonadaceae | 1.511 |  |  |
| 130 | Bacteria.Proteobacteria.Betaproteobacteria.Burkholderiales.Comamonadaceae.Acidovorax | 0.824 |  |  |
| 131 | Bacteria.Proteobacteria.Betaproteobacteria.Burkholderiales.Comamonadaceae.Pelomonas | 1.512 |  |  |
| 132 | Bacteria.Proteobacteria.Betaproteobacteria.Burkholderiales.Oxalobacteraceae | 1.395 |  |  |
| 133 | Bacteria.Proteobacteria.Betaproteobacteria.Burkholderiales.Oxalobacteraceae.Cupriavidus | 3.114 |  |  |
| 134 | Bacteria.Proteobacteria.Betaproteobacteria.Burkholderiales.Oxalobacteraceae.Herbaspirillum | 1.046 |  |  |
| 135 | Bacteria.Proteobacteria.Betaproteobacteria.Burkholderiales.Oxalobacteraceae.Ralstonia | 1.848 |  |  |
| 136 | Bacteria.Proteobacteria.Deltaproteobacteria | 1.551 |  |  |
| **137** | **Bacteria.Proteobacteria.Deltaproteobacteria.Desulfovibrionales** | **5.546** | **4.85997** | **0 mg/kg CTP** |
| **138** | **Bacteria.Proteobacteria.Deltaproteobacteria.Desulfovibrionales.Desulfovibrionaceae** | **5.532** | **4.88636** | **0 mg/kg CTP** |
| 139 | Bacteria.Proteobacteria.Deltaproteobacteria.Desulfovibrionales.Desulfovibrionaceae.Desulfovibrio | 4.23 |  |  |
| 140 | Bacteria.Proteobacteria.Deltaproteobacteria.Desulfuromonadales | 0.766 |  |  |
| 141 | Bacteria.Proteobacteria.Deltaproteobacteria.Desulfuromonadales.Geobacteraceae | 0.766 |  |  |
| 142 | Bacteria.Proteobacteria.Deltaproteobacteria.Desulfuromonadales.Geobacteraceae.Geobacter | 0.766 |  |  |
| 143 | Bacteria.Proteobacteria.Gammaproteobacteria | 2.225 |  |  |
| 144 | Bacteria.Proteobacteria.Gammaproteobacteria.Enterobacteriales | 3.76 |  |  |
| 145 | Bacteria.Proteobacteria.Gammaproteobacteria.Enterobacteriales.Enterobacteriaceae | 3.757 |  |  |
| 146 | Bacteria.Proteobacteria.Gammaproteobacteria.Enterobacteriales.Enterobacteriaceae.Enterobacter | 1.395 |  |  |
| 147 | Bacteria.Proteobacteria.Gammaproteobacteria.Enterobacteriales.Enterobacteriaceae.Escherichia | 0.717 |  |  |
| 148 | Bacteria.Proteobacteria.Gammaproteobacteria.Enterobacteriales.Enterobacteriaceae.Shigella | 1.507 |  |  |
| 149 | Bacteria.Proteobacteria.Gammaproteobacteria.Pseudomonadales | 1.046 |  |  |
| 150 | Bacteria.Proteobacteria.Gammaproteobacteria.Pseudomonadales.Moraxellaceae | 1.046 |  |  |
| 151 | Bacteria.Proteobacteria.Gammaproteobacteria.Pseudomonadales.Moraxellaceae.Acinetobacter | 1.046 |  |  |
| 152 | Bacteria.Tenericutes | 3.633 |  |  |
| 153 | Bacteria.Tenericutes.Mollicutes | 3.633 |  |  |
| 154 | Bacteria.Tenericutes.Mollicutes.Anaeroplasmatales | 2.315 |  |  |
| 155 | Bacteria.Tenericutes.Mollicutes.Anaeroplasmatales.Anaeroplasmataceae | 2.315 |  |  |
| 156 | Bacteria.Tenericutes.Mollicutes.Anaeroplasmatales.Anaeroplasmataceae.Anaeroplasma | 2.315 |  |  |
| 157 | Bacteria.Tenericutes.Mollicutes.RF39 | 3.612 |  |  |
| 158 | Bacteria.TM7 | 1.781 |  |  |
| 159 | Bacteria.TM7.TM7_3 | 1.781 |  |  |
| 160 | Bacteria.TM7.TM7_3.CW040 | 1.781 |  |  |
| 161 | Bacteria.TM7.TM7_3.CW040.F16 | 1.781 |  |  |
| 162 | Bacteria.Verrucomicrobia | 4.085 |  |  |
| 163 | Bacteria.Verrucomicrobia.Verruco_5 | 0.717 |  |  |
| 164 | Bacteria.Verrucomicrobia.Verruco_5.WCHB1_41 | 0.717 |  |  |
| 165 | Bacteria.Verrucomicrobia.Verruco_5.WCHB1_41.RFP12 | 0.717 |  |  |
| 166 | Bacteria.Verrucomicrobia.Verrucomicrobiae | 4.085 |  |  |
| 167 | Bacteria.Verrucomicrobia.Verrucomicrobiae.Verrucomicrobiales | 4.085 |  |  |
| 168 | Bacteria.Verrucomicrobia.Verrucomicrobiae.Verrucomicrobiales.Verrucomicrobiaceae | 4.085 |  |  |
| 169 | Bacteria.Verrucomicrobia.Verrucomicrobiae.Verrucomicrobiales.Verrucomicrobiaceae.Akkermansia | 4.085 |  |  |

Data are presented as the mean and analyzed via a Wilcoxon test (n=5 for vehicle-treated group and n=4 for CTP-treated group). The abundance of dominant nodes was provided with logarithm (log_10_). When there were significant differences between two groups and LDA score > 2, the LDA score and group information were provided.

**
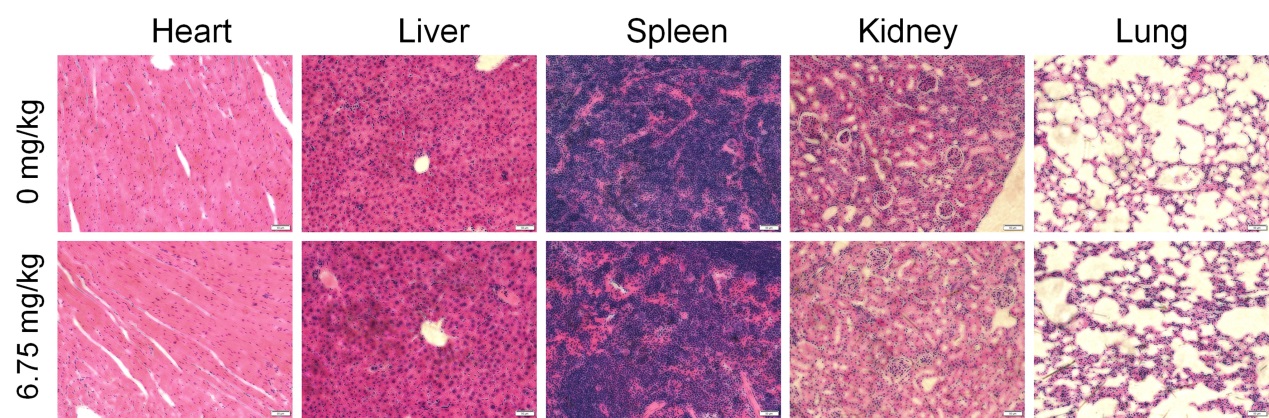
**

**Supplementary Figure S1** Histopathological observation of *Apc*^Min/+^ mice heart, liver, spleen, kidney and lung (200×, Scale bar: 50 μm).


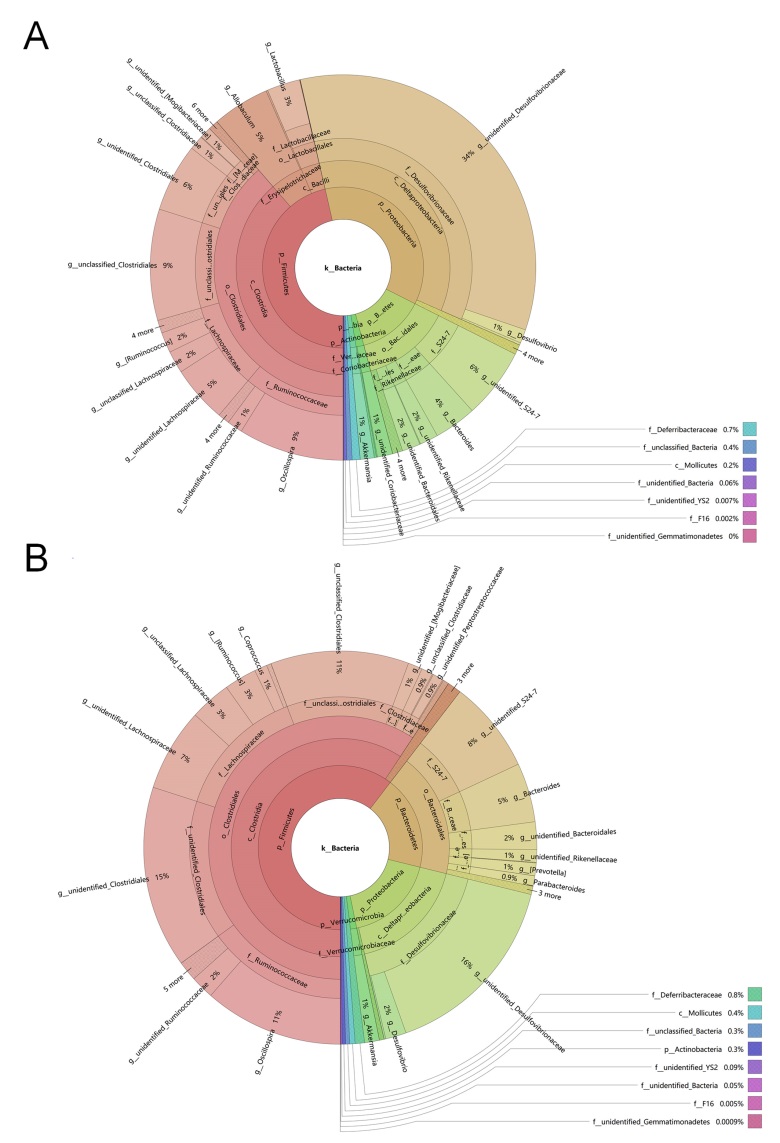


**Supplementary Figure S2** Krona species composition map of intestinal flora in (A) vehicle-treated mice and (B) CTP-treated mice were analyzed with KronaTools (v2.7).
